# Inhibition of Glycogen Synthase Kinase-3β (GSK-3β) as potent therapeutic strategy to ameliorates L-dopa-induced dyskinesia in 6-OHDA parkinsonian rats

**DOI:** 10.1038/srep23527

**Published:** 2016-03-21

**Authors:** Cheng-long Xie, Jing-Ya Lin, Mei-Hua Wang, Yu Zhang, Su-fang Zhang, Xi-Jin Wang, Zhen-Guo Liu

**Affiliations:** 1 Department of Neurology, Xinhua Hospital affiliated to the Medical School of Shanghai Jiaotong University, 200092, 1665 Kongjiang Road, Shanghai, China

## Abstract

Levodopa (L-dopa) is the dominating therapy drug for exogenous dopaminergic substitution and can alleviate most of the manifestations of Parkinson’s disease (PD), but long-term therapy is associated with the emergence of L-dopa-induced dyskinesia (LID). Evidence points towards an involvement of Glycogen Synthase Kinase-3β (GSK-3β) in development of LID. In the present study, we found that animals rendered dyskinetic by L-dopa treatment, administration of TDZD8 (2mg/kg) obviously prevented the severity of AIM score, as well as improvement in motor function (P < 0.05). Moreover, the TDZD8-induced reduction in dyskinetic behavior correlated with a reduction in molecular correlates of LID. TDZD8 reduced the phosphorylation levels of tau, DARPP32, ERK and PKA protein, which represent molecular markers of LID, as well as reduced L-dopa-induced FosB mRNA and PPEB mRNA levels in the lesioned striatum. In addition, we found that TDZD8 antidyskinetic properties were overcome by D1 receptor, as pretreatment with SKF38393 (5 mg/kg, 10 mg/kg, reapectively), a D1 receptor agonist, blocked TDZD8 antidyskinetic actions. This study supported the hypothesis that GSK-3β played an important role in the development and expression of LID. Inhibition of GSK-3β with TDZD8 reduced the development of ALO AIM score and associated molecular changes in 6-OHDA-lesioned rats.

Levodopa (L-dopa) is the dominating therapy drug for exogenous dopaminergic substitution and can alleviate most of the manifestations of Parkinson’s disease (PD). Actually, bradykinesia and other characteristic motor manifestations of PD are relieved by treatment with L-dopa^1^. However, L-dopa–induced dyskinesia (LID) is an almost inevitable debilitating side effects result of long-term L-dopa treatment. Approximately 50% of the patients are affected after 5 years of treatment and almost all patients suffered LID after 10 years^2^. Moreover, LID contributes to severely impair quality of life and raise management costs of PD^3^. Once LID has developed in patients, they are hard to control and management. Young age of PD onset, disease severity, and high doses of L-dopa increase the risk of development of LID^4^. Although more and more evidences emerging from previous studies, their pathogenesis is still unclear and unable to give conclusive answers to questions such as what the pivotal mechanism underlying LID is or which treatment method is the most effective to avoid and ameliorate these dyskinesia^5^.

Recently, a number of studies suggested that treatment with L-dopa at high concentrations increased Glycogen Synthase Kinase-3 (GSK-3) activity and finally resulted in neuronal injury^6^. What is more, previous study reported that bilateral models of PD as MPTP-treated (1-methyl-4-phenyl-1,2,3,6-tetrahydropyridine) monkeys, increased activation of GSK-3β was associated with L-dopa-induced dyskinesias^7^. Evidence points toward an involvement of GSK-3β in development of LID. On the other hand, it was also confirmed in our previous study that tau activation played a critical role in the molecular and behavioural induction of LID in 6-OHDA-lesioned (hydroxydopamine) rats^8^. Another significant finding is that intermittent L-dopa treatment positively correlates with hyper-phosphorylation of the protein tau^9^.

Emerging evidences *in vivo* and *in vitro* have come to further support the unique, crucial position of GSK-3β in the pathogenesis of AD, especially is the major kinase phosphorylating tau protein, triggering cytoskeleton destabilization, tau aggregation, and neuronal death^10^. However, in animal models of L-dopa-induced dyskinesia, there is still limited data on GSK-3β expression involve in development of LID. In the present study, therefore, was mainly to investigate whether GSK-3β inhibitor against AIM score in a rat model of LID. We used selective GSK-3β inhibitor TDZD8 (4-Benzyl-2-methyl-1,2,4-thiadiazolidine-3,5-dione) to explore the involvement of GSK-3β in protein tau phosphorylation. In parallel, we investigated the molecular mechanisms associated with the antidyskinetic effects of TDZD8 (1 mg/kg and 2 mg/kg) and assessed the occurrence of AIM score and the performance of motor function, as well as PKA activity and/or DARPP-32, ERK phosphorylation in the striatum of LID rats.

## Results

### Effects of L-dopa on GSK-3β and CDK5 in 6-OHDA-lesioned rats

**Figure 1:**
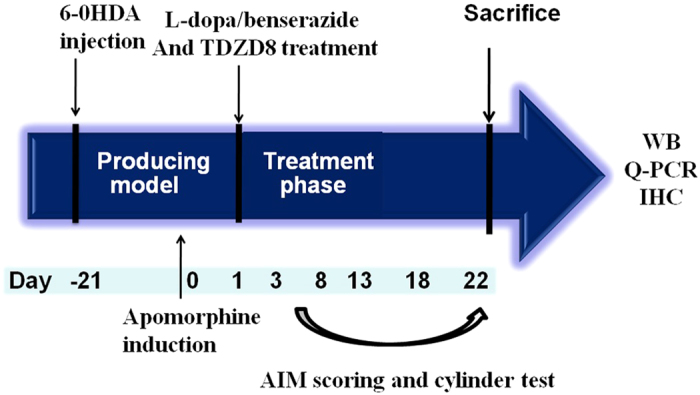
The protocol of the experiment. SD rats (36) were unilaterally injected with 6-OHDA in the medial forebrain bundle (MFB). Contralateral turning behaviours after apomorphine injection were tested 21 days later. Apomorphine-induced rotations exceeded 7 turns/min were enrolled in the next experiment. Rats in the LID group (n = 8) were administrated once-daily (9:00 am) with L-dopa (25 mg/kg, i.p.) plus benserazide (6.25 mg/kg, i.p.) for 3 weeks; Rats in the TDZD8 groups were further divided into two groups, namely TDZD8 low dose group (TDZD8-L, 1 mg/kg, n = 8) and TDZD8 high dose group (TDZD8-H, 2 mg/kg, n = 8). Rats received TDZD8 also one time per day for 3 weeks. Additionally, rats in the PD group (n = 8) and sham group (n = 4) were treated physiological saline for 3 weeks. AIMs were evaluated during this period at days 2, 7, 12, 17 and 21. The animals were sacrifced 2 h after the last injection for western blot and Q-PCR. TDZD8: 4-Benzyl-2-methyl-1,2,4-thiadiazolidine-3,5-dione; i.p.: intraperitoneal.

**Figure 2:**
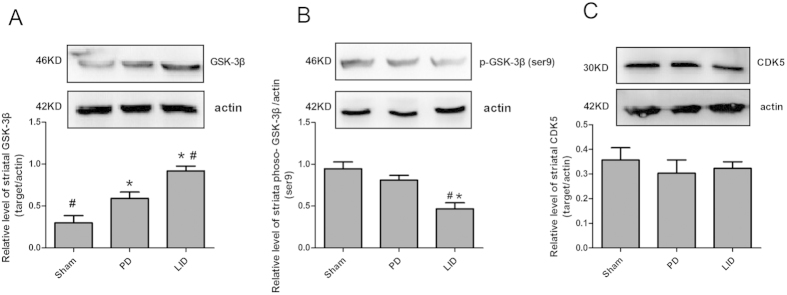
Protein levels were evaluated by Western blotting of proteins extracted from the striatum ipsilateral to the 6-OHDA lesion. They were assessed in extracts from sham (n = 4) or 6-OHDA-lesioned rats treated with vehicle (n = 4), pulsatile L-dopa (25 mg/kg) plus benserazide (6.25 mg/kg, n = 4) on day 23. (**A**) GSK-3β levels expressed relative to actin levels; (**B**) Phosphorylated GSK-3β (ser9) levels expressed relative to actin levels; (**C**) CDK5 levels expressed relative to actin levels. The data represent the mean of relative optical density ± SD; *p < 0.05 vs sham group; ^#^p < 0.05 vs PD group (one-way ANOVA followed by LSD post-hoc analysis).

44 rats were unilaterally injected with 6-OHDA in the MFB (medial forebrain bundle) except sham group. Figure 1 described the experimental design. Those treated with L-dopa plus benserazide (LID group, n = 4) were showed high levels of GSK-3β and low level of phoso-GSK-3β (ser9) when compared with 6-OHDA lesioned rats (PD group, n = 4) and normal control rats (Sham groups, n = 4) in the striatum lysis (P < 0.05 compared with PD groups and Sham groups, Fig. 2A,B). On the contrary, the protein for CDK5 was evenly expressed in the corpus striatum, namely, there was no overall difference in the level of CDK5 in the Sham, PD and LID groups, respectively (P > 0.05 compared with PD group and Sham group, Fig. 2C).

### Treatment with TDZD8 prevents the development of LID in 6-OHDA-lesioned rats

**Figure 3:**
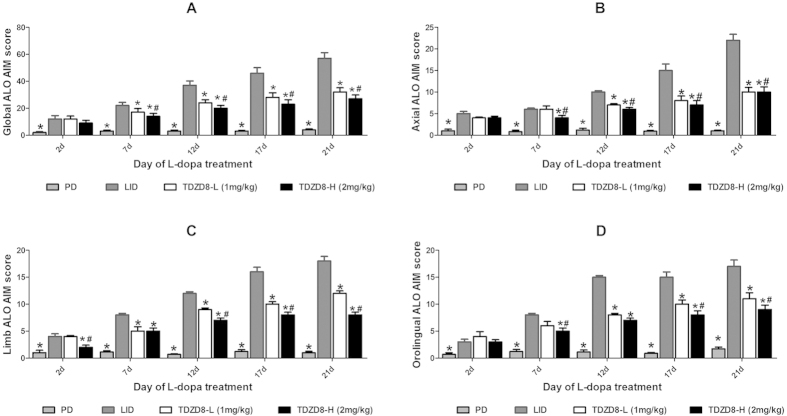
The effect of TDZD8 on the development of L-dopa-induced dyskinesia in 6-OHDA-lesioned rats. Chronic TDZD8 treatment significantly attenuates dyskinesia score for 3 weeks. For each day, a total AIM score was calculated as the sum of the basic score multiplied by the amplitude score for each AIM subtype, excluding the locomotive subtype. (**A**) time course of the sum of axial, limb, and orolingual (**B**) timecourse of the axial score, (**C**) time course of the limb score and (**D**) time course of the orolingual score on each testing session were rated following the administration of L-dopa and TDZD8. Data are presented as mean ± SD. *p < 0.05 vs LID group; ^#^p < 0.05 vs TDZD8-L group (Kruskal Wallis followed by Dunn’s test for multiple comparisons or a Mann–Whitney U test).

PD rats treated with L-dopa for 21 days developed a progressive increase in LID as indicated by ALO AIM score (p < 0.05 for treatment effect, p < 0.05 for time effect and p < 0.01 for treatment and time interaction, Fig. 3A). As expected, the PD group received the saline for 21 days did not develop LID features (Fig. 3A). Meanwhile, co-administration of TDZD8, an inhibitor of GSK-3β, with L-dopa did not develop severe LID over the 21 day treatment period, which differed significantly from the LID group in all testing sessions except at 2 day time point after administration (p < 0.05, Fig. 3A). Furthermore, TDZD8-H (2 mg/kg, intraperitoneal injection (i.p.)) group demonstrated certain more reduction in the ALO AIM score compared with the rats receiving TDZD8-L (1 mg/kg, i.p.), but the rats still showed a mild dyskinesia, this effect did not reach completely reversed. Similarly, this seemed to be the same trend in Axial AIM (Fig. 3B), limb AIM (Fig. 3C) as well as orolingual AIM (Fig. 3D).

### Effect of TDZD8 on parkinsonion disability score

**Figure 4:**
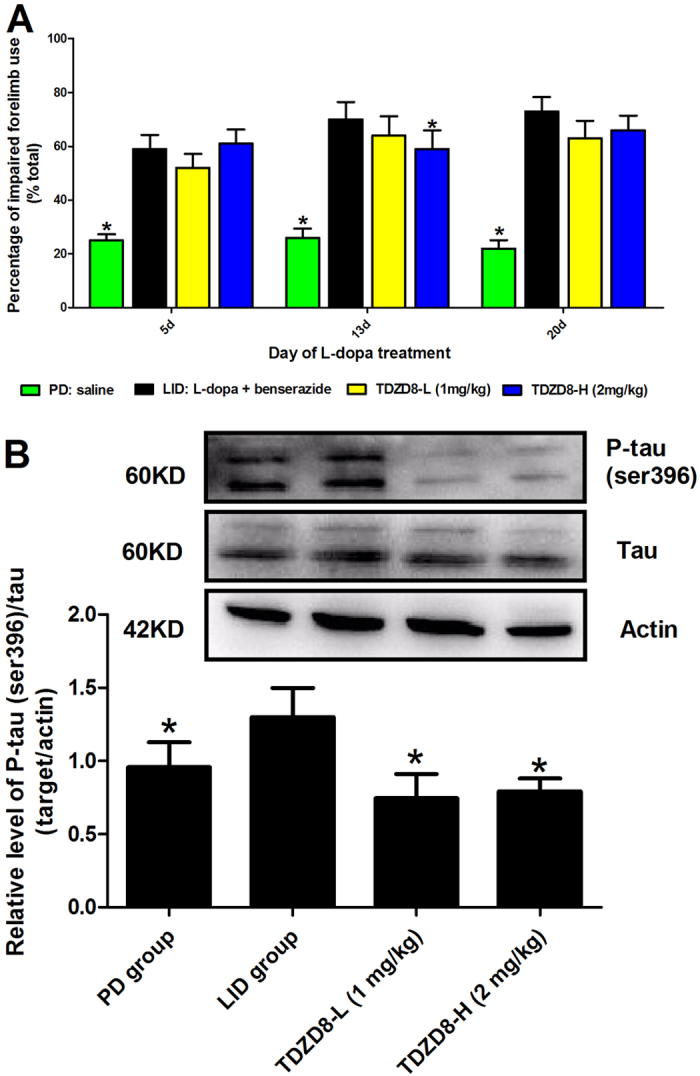
(**A**) Unilaterally 6-OHDA-lesioned rats were tested for the percentage of impaired forelimb use compared with the total numbers. Animals were injected with saline, L-dopa (25 mg/kg) plus benserazide (6.25 mg/kg), TDZD8-L (1 mg/kg) or TDZD8-H (2 mg/kg). Motor effects of TDZD8 in 6-OHDA-lesioned animals showed the chronic administration of TDZD8 could significantly improve parkinsonion disability score. Experiment consisted of three different sessions, namely 5d, 13d and 20d in the treatment period. Data are presented as mean ± SD. *p < 0.05 vs LID group, n = 8 per group; (**B**) Phosphorylated levels of tau (ser396) induced by L-dopa administration in 6-OHDA-lesioned animals was analyzed by Western blot in striatal samples. TDZD8 prevented increased level of p-tau and total tau after pulsatile L-dopa treatment. They were assessed in extracts from sham or 6-OHDA-lesioned rats treated with vehicle, pulsatile L-dopa plus benserazide or TDZD8-L (1 mg/kg) and TDZD8-H (2 mg/kg). The data represent the mean of relative optical density ± SD. *p < 0.05 vs LID group (one-way ANOVA, n = 4).

We next sought to determine whether the phenomenon of TDZD8 attenuation of LID without ablation of the therapeutic response to L-dopa. Consequently, we co-administered TDZD8 (1 mg/kg, 2 mg/kg, respectively) with L-dopa to 6-OHDA-lesioned rats for 21 days. We observed that 6-OHDA-lesioned rats treated with L-dopa prefer to use the compromised (contralateral) forelimb to touch the inner wall of the cylinder compared with the 6-OHDA-lesioned rats treated with saline (p < 0.05, Fig. 4A). If the animals were co-injected with TDZD8-L (1 mg/kg) or TDZD8-H (2 mg/kg) for 21 days, they also demonstrated preferential to touch the wall with contralateral forelimb at 5, 13 and 20 day time points after administration (p > 0.05 compared with LID group, p < 0.05 compared with PD group, Fig. 4A). When we continued to measure forelimb preference between TDZD8-L group and TDZD8-H group, we found that it was no significant difference between two groups (p > 0.05, Fig. 4A). Namely, TDZD8-H tested (2 mg/kg) did not produce an additional benefit over the TDZD8-L (1 mg/kg) administration in terms of forelimb functional test.

### Treatment with TDZD8 prevents phosphorylation of tau, Tyrosine hydroxylase (TH), DARPP32, ERK and PKA protein

**Figure 5:**
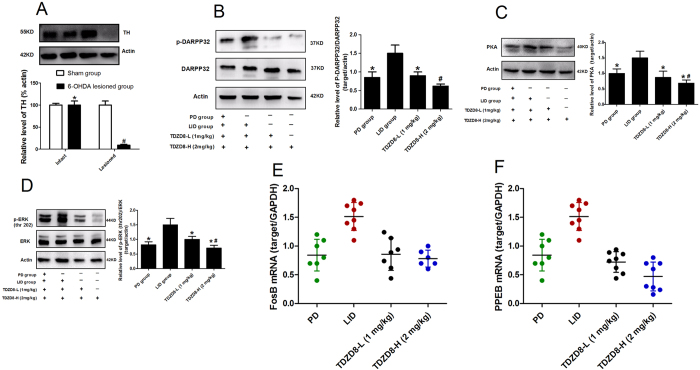
(**A–D**) Protein levels were evaluated by Western blotting of proteins extracted from the striatum ipsilateral to the 6-OHDA lesion. They were assessed in extracts from 6-OHDA-lesioned rats treated with vehicle, pulsatile L-dopa (25 mg/kg) plus benserazide (6.25 mg/kg), TDZD8-L (1 mg/kg) and TDZD8-H (2 mg/kg). (**A**) The degree of dopamine depletion induced by 6-OHDA lesions and sham. Tyrosine hydroxylase (TH) levels expressed relative to actin levels. ^#^p < 0.01 and *p > 0.05 vs sham-intact hemisphere (n = 4); (**B**) phosphorylated DARPP32 level expressed relative to tatal DARPP32 level; (**C**) PKA level expressed relative to actin levels; (**D**) phosphorylated ERK1/2 level expressed relative to total ERK1/2 level; (**E,F**) mRNA expression of FosB and PPEB were evaluated by Q-PCR from the striatum ipsilateral to the 6-OHDA lesion. (**E**) FosB mRNA level expressed relative to GAPDH mRNA; (**F**) PPEB mRNA level expressed relative to GAPDH mRNA. *p < 0.05 vs LID group; ^#^p < 0.05 vs TDZD8-L group (one-way ANOVA, n = 4 per group).

Previous studies had documented that phosphorylated level of tau (ser396) in the striatum of LID group was mild increase compared with PD group^8^. In this study, we confirmed that this elevation in p-tau level was apparent prevented in animals treated with TDZD8 (p < 0.05, Fig. 4B). Meanwhile, we observed a dose dependent response between TDZD8-L and TDZD8-H for inhibited the up-regulation of p-tau (p < 0.05 compared with TDZD8-L group, Fig. 4B). The degree of dopamine depletion was verified by western blotting with an antibody raised against TH. We found no significant changes were observed in TH levels in the intact hemisphere between sham-operated and 6-OHDA-lesioned rats (p > 0.05, Fig. 5A). In contrast, TH level was obviously decreased by more than 92% in the lesioned side of 6-OHDA-injected rats when compared with sham group (p < 0.001, Fig. 5A). Figure 5B showed that chronic L-dopa treatment of hemiparkinsonian rats increased phosphorylation of DARPP32 in the lesioned striatum. TDZD8 at 1 mg/kg or 2 mg/kg both reduced the induction of p-DARPP32 following chronic L-dopa treatment in parkinsonian animals. Furthermore, TDZD8-H (2 mg/kg) showed more reduction in terms of p-DARPP32 level compared with TDZD8-L administration (1 mg/kg, p < 0.05, Fig. 5B). Interestingly, TDZD8 reduced the level of the p-DARPP32 below control group. However, we found no significant statistical difference between TDZD8 and PD group (p > 0.05, Fig. 5B). Similarly, repeated administration of L-dopa in hemiparkinsonian animals greatly increased PKA level. In keeping with previous results, TDZD8 treatment for 21d of rats with established dyskinesia also induced a significant reduction in PKA expression (p < 0.05 compared with LID group), observed co-administration with L-dopa (25 mg/kg) in a dose dependent manner (p < 0.05 compared with TDZD8-L group, Fig. 5C). Meanwhile, although TDZD8 group showed more reduction level of PKA protein than control, no statistical difference was obtained between TDZD8 group and PD group (p > 0.05, Fig. 5C). In accordance with aforementioned results, we found that acute L-dopa treatment induced a marked increase in p-ERK in the lesioned hemispheres compared with PD group (p < 0.05, Fig. 5D). TDZD8 treatment significantly reduced the chronic L-dopa-induced ERK phosphorylation in the striatum relative to the LID group (p < 0.05, Fig. 5D). Interestingly, the expression of p-ERK was also more significantly reduced in response to TDZD8-H than TDZD8-L administration (p < 0.05 compared with TDZD8-L, Fig. 5D). Furthermore, we pooled the whole data to process and found no significant difference when TDZD8 group compared with PD group according to p-ERK (p > 0.05, Fig. 5D).

### Effects of TDZD8 on FosB and PPEB mRNA

As reported in a number of previous studies that FosB mRNA level was significantly increased ipsilaterally to the lesion side in the LID group after treatment with L-dopa (p < 0.05 compared with PD group, Fig. 5E). However, co-administration of TDZD8 showed an approximate 40% reduction in FosB mRNA level in the striatum (p < 0.05 vs LID group, Fig. 5E). Dose dependent curve was also observed in the FosB mRNA level. However, we found that it was no significant difference between TDZD8-L and TDZD8-H groups (p > 0.05 compared with TDZD8-L group, Fig. 5E). Meanwhile, we also compared striatal PPEB mRNA among the four groups and found treatment with L-dopa can apparent rise the PPEB mRNA level compared with other three groups (p < 0.05, Fig. 5F). In accordance with previous results, treatment with TDZD8 lowered the expression of PPEB mRNA to similar levels as in 6-OHDA-lesioned rats without treated with L-dopa (p > 0.05, Fig. 5F).

### The anti-dyskinetic effect of TDZD8 is overcome by D1Rs agonist

**Figure 6:**
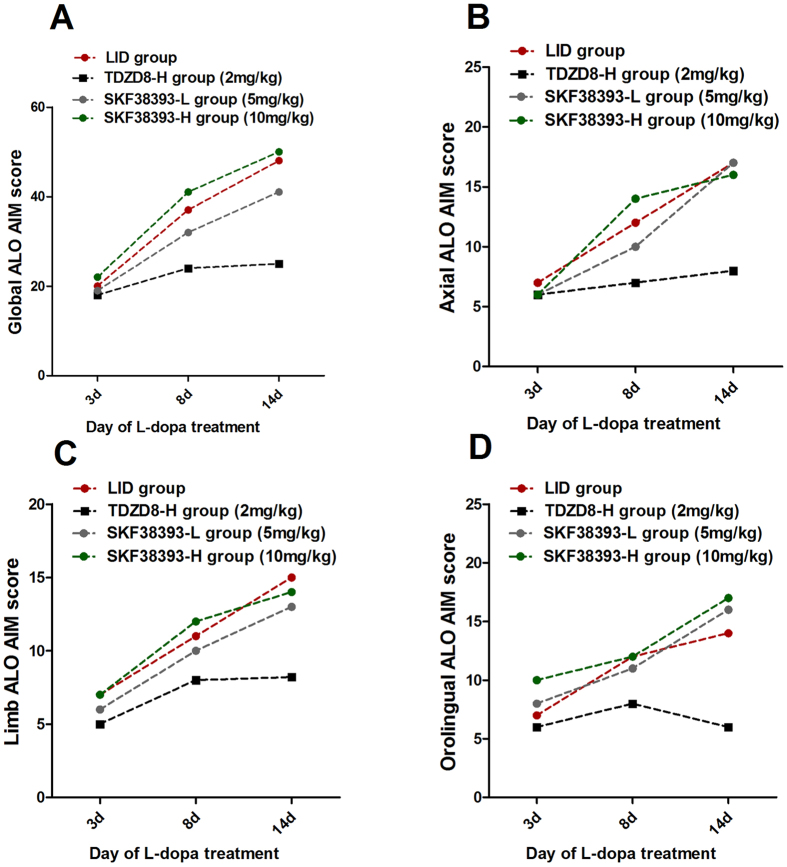
The decrease in dyskinesia induced by TDZD8 is overcome by dopamine rceptor-1 agonist. Treatment with SKF38393 (D1 receptor agonist, 5 mg/kg or 10 mg/kg) abolished the antidyskinetic effect of TDZD8, either individual dyskinetic subtypes; (**A**) global dyskinetic score; (**B**) axial AIM score, (**C**) forelimb AIM score and (**D**) orolingual AIM score. Data are presented as mean ± SD (Kruskal Wallis followed by Dunn’s test for multiple comparisons or a Mann–Whitney U test).

From previous certain studies we know that some of the major abnormalities related to sensitized D1R-signaling and associated to LID. To further test whether the D1Rs has any role in the antidyskinetic effect of TDZD8, SKF38393 (5 mg/kg or 10 mg/kg), a D1R agonist, was injected 15 min before TDZD8. ALO AIMs were measured evaluated during this period at days 3, 8 and 14 after initiated L-dopa administration (Fig. 6). Among them, 6-OHDA-lesioned rats treated with L-dopa plus benserazide for 14 days developed a progressive increase in LID. Median ALO AIM score increased from 20.6 ± 3.18 on day 3 to 48.5 ± 6.02 on day 14 (n = 4, P < 0.05 vs day 2, Fig. 6A). Meanwhile, co-administration of 2 mg/kg TDZD8 with L-dopa did not develop severe LID over the 14 day treatment period, which differed significantly from the LID group in all testing sessions except at 2 day time point after administration (p < 0.05, Fig. 6A). Treatment with 5 mg/kg or 10 mg/kg SKF38393 significantly blocked the anti-dyskinetic effects of TDZD8 as observed for Global ALO AIM score (p < 0.05 vs TDZD8-H group at day 8 and 14, Fig. 6A). Higher doses of SKF38393 (10 mg/kg) were more effective for offset the anti-dyskinetic effects of TDZD8 than lower doses of SKF38393 (5 mg/kg, p < 0.05 vs SKF38393-L group, Fig. 6A). This seemed to be the nearly same trend in axial AIM (Fig. 6B), limb AIM (Fig. 6C) as well as orolingual AIM (Fig. 6D).

## Discussion

We used a hemiparkinsonian rat’s model of dyskinesia based on unilateral 6-OHDA striatal lesion, followed by chronic L-dopa administration of a daily dose of 25mg/kg. In this model animals progressively develop dyskinetic symptoms, including axial, forelimb and orolingual dyskinesias, namely ALO AIMs. The major findings from this study were: (1) TDZD8 reduced L-dopa induced dyskinesia; (2) the effective TDZD8 dose (1 mg/kg or 2 mg/kg) did not interfere with the therapeutic motor effects of L-dopa; (3) TDZD8 reduced L-dopa-induced p-tau, p-DARPP32, p-ERK and PKA over-expression in lesioned striatum; (4) TDZD8 reduced L-dopa-induced FosB mRNA and PPEB mRNA levels in the lesioned striatum; (5) anti-dyskinetic actions of TDZD8 were overcome by D1R agonist. Our results showed significant antidyskinetic properties of TDZD8. The anti-dyskinetic effect of the 2 mg/kg dose of TDZD8 was observed 7d after L-dopa administration, when dyskinesias were occurring. Moreover, this effect of TDZD8 was maintained over the entire 21 days chronic administration protocol without development of tolerance. Our findings also showed that TDZD8 reduced D1R-dependent signaling, as demonstrated by a decrease in the molecular markers after L-dopa treatment such as DARPP32, PKA, ERK and FosB *et al.*

In the present study, we confirmed a dose range of TDZD8 that is effective in reducing AIM score. We found co-administration of TDZD8 with L-dopa did not develop severe LID over the 21 day treatment period, while TDZD8-H group demonstrated certain more reduction in the ALO AIM score compared with the rats receiving TDZD8-L. In terms of anti-parkinsonian action, TDZD8 did not alter L-dopa’s efficacy in improving impaired forelimb compared with the total number of limb use movements in lesioned animals to exclude the possibility that the anti-dyskinetic efficacy of TDZD8 was due to an attenuation of L-dopa’s efficacy. Recently, some researches lend support to the view that activation of striatal GSK3 signaling was proposed to play a role in the mechanisms implicated in development of LID in a rodent model of PD^11^. Moreover, Morissette *et al.* showed that in MPTP-treated monkeys, loss of striatal dopamine increased GSK-3β activation was associated with LID development^7^. Which was consistent with our results that treatment with L-dopa in rats chronically were showed high levels of GSK-3β when compared with 6-OHDA-lesioned rats and normal control rats in the striatum lysis. Furthermore, we had shown that in 6-OHDA-lesioned rats where L-dopa plus TDZD8 treatment preventing LID occur and development, as well as substantial decrease of phoso-tau level in the striatum. It was likely that enhanced GSK-3β and tau activity by L-dopa were offset by the TDZD8 administration.

To our knowledge, it is now well established that 6-OHDA-lesioned medial forebrain bundle results in the enhanced responsiveness of striatal neurons of the direct striatonigral pathway after intermittent L-dopa administration^12^. Emerging evidence indicates that increased responsiveness of the D1R results from chronically L-dopa administration results in augmented synthesis of cAMP and hyper-activation of PKA and DARPP-32^9^. In addition, pharmacological inhibition of PKA, or genetic inactivation of DARPP-32 have been shown to prevent LID occur and development. Meanwhile, abnormal PKA/DARPP-32 signaling increases the phosphorylation of ERK, which controls transcriptional and translational processes^13^. In the nucleus, PKA/DARPP-32 and ERK signaling leads to increased expression of immediate early genes (FosB) and prodynorphin (PPEB)^14^. In the present study, we demonstrated that GSK-3β inhibition with TDZD8 totally prevented the L-dopa-induced increase in expression of phosphorylation levels of DARPP-32 and ERK1/2, as well as PKA activation in the striatum. It is suggested that the persistent hyperactivation of D1R signaling by L-dopa administration in 6-OHDA-lesioned rat model of PD, which leads to GSK-3β activation, might be contributes to development of LID. Accordingly to this idea, our results showed that concomitant administration of GSK-3β antagonist and L-dopa entirely prevented up-regulation of the main marker (DARPP32/PKA/ERK) of LID that was associated with behavioural sensitization. Consequently, these data indicated that GSK-3β played a pivotal role in molecular priming for dyskinesia by L-dopa and aberrant D1-dependent molecular plasticity in the striatum.

Another key finding in this study was that L-dopa treatment positively correlated with hyperactivation of the FosB and PPEB levels and prevented by TDZD8 administration. Based on previous studies, it has been established several times that LID show a high striatal expression of PPEB mRNA and transcription factor FosB^15^, which are well-established molecular markers of LID in both rat and non-human primate models of PD^16^. The increase in FosB associated to LID is restricted to the D1R-expressing MSNs of the direct pathway and is mediated via activation of the D1R/cAMP cascade, which is strongly induced by administration of L-dopa^17^. Not only that, studies performed in 6-OHDA-lesioned rats showed that LID was reduced by striatal injection of a FosB antisense oligonucleotide^18^. Moreover, in the same animal model, it has been recently shown that viral vector-induced overexpression of ΔFosB enhances the ability of L-dopa to induce dyskinetic behavior^19^. In fact, our experiment demonstrated that TDZD8 reduced L-dopa-induced FosB mRNA and PPEB mRNA levels in the lesioned striatum. These data indicated that inactivation of GSK-3β prevented molecular adaptation from occurring and supported the hypothesis that GSK-3β played a pivotal role in molecular priming for LID through D1-dependent direct pathway.

Our results indicated that SKF38393 (10 mg/kg) significantly blocked the anti-dyskinetic effects of TDZD8 as observed for each of the dyskinetic symptoms individually as well as in the total dyskinetic score. These results indicated that the stimulation of D1 receptors with SKF39393 specifically abolished the anti-dyskinetic effects of TDZD8. In accordance with aforementioned results and speculated that GSK-3β played a pivotal role in molecular priming for LID probable through D1-dependent direct pathway. In addition, the results were only indicating that an overstimulation of D1R can overcome the protective effects of TDZD in dyskinesia. Therefore, another explanation is that SKF38393 and TDZD8 could be affecting AIMs in the opposite directions completely independently but the effect of SKF38393 was just stronger. Consequently, the results should be interpreted with caution. Moreover, Lebel *et al.* reported Dopamine D1 receptor activation induces tau phosphorylation via GSK3 signaling pathways^20^. In the literature, stimulation of D1 receptors increased GSK-3β activity leading to tau hyperphosphorylation^21^, indicating a role for D1R involvement in the activation of GSK-3β, which should be verified by future studies.

## Conclusions

This study supports the hypothesis that GSK-3β plays an important role in the development and expression of LID. Inhibition of GSK-3β with TDZD8 reduces the development of ALO AIM score and associated molecular changes in 6-OHDA-lesioned rats through a D1R-dependant pathway.

## Materials and Methods

### Animals

Experiments were conducted on sixty female Sprague-Dawley (SD) rats (Shanghai, people republic of China; weight 180–220 g). Upon their arrival, the animals were housed in clean cages with a maximum of five rats per cage under a 12 h light:12 h dark cycle, temperature 22.0 ± 2.0 °C and relative humidity of 55 ± 10%. Food and water were available ad libitum and animal care supervised by veterinarians skilled in the health care center. All experimental protocols involving the animals were reviewed and approved by the Ethical Committee of the Medical School of Shanghai Jiaotong University. The methods were carried out in accordance with the approved guidelines and regulations of the National Institutes of Health for the care and use of laboratory animals.

### Induction of L-dopa-induced dyskinesia (LID)

Unilateral 6-OHDA-lesioned PD models were performed according to our previous studies^8^,^22^. Briefly, rats were deeply anesthetized by 10% chloral hydrate (0.35 ml/100g) and mounted in a stereotaxic apparatus equipped with a rat adaptor. 6-OHDA (32 mg dissolved in 8 μL of 0.9% normal saline containing 0.2% ascorbic acid) was stereotaxically injected into the right MFB of rats. Three week after surgery, the lesioned rats were screened behaviorally using an apomorphine hydrochloride-induced (0.5 mg/kg, i.p.) rotation test and all animals exhibited >7 full body turns/min toward the side of the unlesioned side were selected for the next experiment. Once parkinsonism was stable (3 weeks post 6-OHDA), they were then treated with once-daily administration of L-dopa (25 mg/kg, i.p.) plus benserazide (6.25 mg/kg, i.p.) for 3 weeks to induce a rat model of dyskinesia.

### Drugs and treatment

Apomorphine hydrochloride (Sigma-Aldrich, St. Louis, MO, USA) was administered (0.5 mg/kg). L-dopa (Sigma-Aldrich, 25 mg/kg) plus benserazide-HCl (Sigma-Aldrich, 6.25 mg/kg) were given once-daily. TDZD8, a non-ATP competitive inhibitor of GSK-3β, was dissolved in 10% DMSO and was administered i.p. (TDZD8-L group, 1 mg/kg; TDZD8-H group, 2 mg/kg, respectively) 30 min prior to L-dopa intake for 3 weeks. The dose of TDZD8 used was based on previous studies^23^. (±)-1-Phenyl-2,3,4,5-tetrahydro-(1H)-3-benzazepine-7,8-diol hydrochloride (SKF38393, Abcam, UK), a D1 Dopamine receptor agonist, was dissolved in saline and was administered i.p. (SKF38393-L group, 5 mg/kg; SKF38393-H group, 10 mg/kg, respectively) 30 min prior to L-dopa intake for 3 weeks. The dose of TDZD8 used was based by Iderberg *et al.*^24^. The dose of L -dopa (25 mg/kg) was chosen by our previous study^25^.

### AIM ratings and forelimb functional test

A battery of ALO AIM ratings was performed as our previously described^11^. Briefly, for quantification of LID, rats were observed individually every 20 min from 20 to 120 min. 0 = absent, 1 = present less than 50% of the observation period, 2 = present more than 50% of the observation time, 3 = present all the times but suppressible by external stimuli, and 4 = present all the times and not interfering by external stimuli. For each rat, the maximum theoretical score per monitoring session was 4*3*6 = 72. During a period of 120 min following levodopa treatment, three subtypes of AIM were assessed as axial, limb, orolingual every 20 min (3 min monitoring period for each). The ALO AIM was tested at 2, 7, 12, 17 and 21 days during levodopa treatment. In terms of Parkinsonian disability score, 90 min after levodopa administrated and behavioral assessments were then carried out. A quantitative assessment of locomotor activity using forelimb functional test was performed five times at 5, 9, 13, 16 and 20 days during levodopa treatment, which could as an index of parkinsonion disability score. The rats were placed in a glass cylinder with a diameter of 22 cm and a height of 35 cm to record forelimb use during vertical exploration for 60 min. During a period of 60 min following levodopa treatment, forelimb functional test was assessed every 20 min (3 min monitoring period for each). The final value was expressed in terms of the percentage use of the impaired forelimb (contralateral) compared with the total number of limb use movements^8^,^26^.

### Western blot

Corpus striatum of rats was processed as previously described^8^. Proteins were separated by electrophoresis and transferred overnight to polyvinylidene difluoride membranes. Then, the membrane was incubated with polyclonal rabbit anti-GSK-3β IgG and monoclonal mouse anti-CDK5 IgG (diluted 1:1000; Cell Signaling Technology (CST), USA), polyclonal mouse anti-phospho-tau at ser396 IgG2b and polyclonal rabbit anti-tau IgG (diluted 1:1000; CST; diluted 1:1000; Sigma-Aldrich), monoclonal mouse anti-phospho-ERK at Thr202 IgG and monoclonal rabbit anti-ERK IgG (diluted 1:1000; CST), polyclonal rabbit anti-phospho-DAPRR32 at Thr34 IgG and monoclonal rabbit anti-DARPP32 IgG (diluted 1:1000; Millipore, USA; diluted 1:1000; CST) and polyclonal rabbit phoso-GSK-3β at ser9 (diluted 1:1000; CST) overnight at 4 °C, respectively, and then incubated in horseradish peroxidase conjugated secondary anti-rabbit and anti-mouse β-actin IgG (diluted 1:1000; Beyotime Institute of Biotechnology). The signal was visualized by ECL (A:B 1:1; Millipore) and quantified using Quantity One software (Image Lab).

### Quantitative-Polymerase Chain Reaction (Q-PCR)

Striatal tissues of rats were homogenized and total ribonucleic acid (RNA) was extracted by Trizol reagent (Invitrogen, USA). cDNA was generated from total RNA samples using the RevertAid First Strand cDNA Synthesis kit (Takara, Japan). Q-PCR was performed using the ABI 7500 Real-Time PCR System (Life Technologies, USA) according to the supplier’s instructions. After amplification, melting curve analysis and length verification by gel electrophoresis were carried out to confirm the specificity of PCR products. As a negative control, template RNA was replaced with PCR-grade water. Calculations of threshold cycle and difference were analyzed with ABI 7500 Real-Time PCR System (Life Technologies). Results were expressed as relative expression corrected to the housekeeping gene β-GAPDH. The detector used in real-time PCR reaction is SYBR Green.

The primer sequences used in this study were as follows:

5-GAGAATGAGGTTGCTTTGGAA-3 (forward) and 5-AGACGCTGGTAAGGAGTTGG-3 (reverse) for preproenkephalin B (PPEB) mRNA;

5-GACTCCAGGCGGAAACGGAT-3 (forward) and 5-TCGTAAGGGATCTTGCAGCC-3 (reverse) for FosB mRNA.

### Statistical analysis

Data were expressed as the mean ± standard deviation (SD). The scores assigned for AIM and parkinsonion disability are non-parametric and were analyzed using a Kruskal Wallis followed by Dunn’s test for multiple comparisons in the case of comparing data over multiple days, or a Mann–Whitney U test. The western blot, Q-PCR conformed to normal distribution were performed using one-way analysis of variance (ANOVA) followed by LSD post-hoc comparisons when appropriate as indicated in the figure legends. P-values < 0.05 were considered statistically significant differences. All analyses were carried out using SPSS 16.0.

## Additional Information

**How to cite this article**: Xie, C.- *et al.* Inhibition of Glycogen Synthase Kinase-3β (GSK-3β) as potent therapeutic strategy to ameliorates L-dopa-induced dyskinesia in 6-OHDA parkinsonian rats. *Sci. Rep.* **6**, 23527; doi: 10.1038/srep23527 (2016).
